# University of Pennsylvania—Department of Veterinary Medicine

**Published:** 1895-07

**Authors:** 


### University of Pennsylvania—Department of Veterinary Medicine.

At a public commencement, held Thursday, June 13, 1895, at the American Academy of Music, the degree of Doctor of Veterinary Medicine was conferred by Charles C. Harrison, A.M., Provost, upon the following candidates, after which an address was delivered by Horatio C. Wood, M.D., LL.D., Professor of Materia Medica, Pharmacy, and General Therapeutics, and Clinical Professor of Nervous Diseases: R. Markley Black,

Cecilton, Md.; Charles W. Boyd, Allegheny City, Pa.; Marsh L. Brackbill, Strasburg, Pa.; Frederick L. Felber, Baltimore, Md.; Bert Hagenbuch, Mahanoy City, Pa.; John R. Hart, Philadelphia; Edwin Hogg, Kirkwood, Pa.; Ulysses G. Houck, Berwick, Pa.; Wilmer B. Kille, Masonville, N. J.; J. Stewart Lacock, Allegheny City, Pa.; W. Walter Martin, Philadelphia, Pa.; James M. Mecray, Maple Shade, N. J.; Harry E. Oesterling, Wheeling, W. Va.; William P. Phipps, Lionville, Pa.; William J. Reagan, Philadelphia, Pa.; Joseph C. Robert, B.S., Centreville, Miss.; Willis B. Stauffer, Philadelphia, Pa.; Benjamin M. Underhill, Knoxville, Iowa.

The J. B. Lippincott prize of $100 for the best general average in the three-years' examinations was won by Ulysses G. Houck.

An écraseur offered to students of the second-year class passing the best examination in veterinary anatomy was won by Herman A. Christian.

